# Application of an iterative Bayesian variable selection method in a genome-wide association study of rheumatoid arthritis

**DOI:** 10.1186/1753-6561-1-s1-s109

**Published:** 2007-12-18

**Authors:** Soonil Kwon, Dai Wang, Xiuqing Guo

**Affiliations:** 1 Medical Genetics Institute, Cedars-Sinai Medical Center, 8635 West Third Street, Suite 665, Los Angeles, California 90048, USA

## Abstract

Genome-wide association studies usually involve several hundred thousand of single-nucleotide polymorphisms (SNPs). Conventional approaches face challenges when there are enormous number of SNPs but a relatively small number of samples and, in some cases, are not feasible. We introduce here an iterative Bayesian variable selection method that provides a unique tool for association studies with a large number of SNPs (*p*) but a relatively small sample size (*n*). We applied this method to the simulated case-control sample provided by the Genetic Analysis Workshop 15 and compared its performance with stepwise variable selection method. We demonstrated that the results of iterative Bayesian variable selection applied to when *p *» *n *are as comparable as those of stepwise variable selection implemented to when *n *» *p*. When *n *> *p*, the iterative Bayesian variable selection performs better than stepwise variable selection does.

## Background

Advances in genotyping technology have made genome-wide association studies feasible. Usually, a large number of single-nucleotide polymorphisms (SNPs) are engaged in a genome-wide association study. Many statistical approaches have been used to analyze the genome-wide association data. Conventional statistical approaches, however, face many challenges for analyzing the data in which a relatively small number of samples that are realistic to recruit for a research study contain hundreds of thousands of markers densely spaced over the genome. Various statistical approaches that can be utilized when *p *» *n *have been applied to reduce dimension. West et al. [1] utilized singular value decomposition in the design matrices of Bayesian regression analysis with binary responses. Sha et al. [2] applied stochastic search variable selection, which is a Bayesian variable selection (BVS) approach proposed by George and McCulloch [3], to identify molecular signatures of disease stage.

Although shown to be very promising, BVS uses quite long iterations and take a long time to search for significant SNPs. In order to overcome these problems, we propose an iterative Bayesian variable selection (IBVS) method, which repeatedly uses the BVS with relatively small iterations until a proper number of SNPs are selected. We applied the IBVS to randomly selected sub-samples of the simulated rheumatoid arthritis (RA) data provided by the Genetic Analysis Workshop 15 (GAW15) Problem 3 to find subsets of SNPs that are associated with RA status. The results obtained by using IBVS were compared to those obtained from stepwise variable selection (SVS) to evaluate the validity and performance of IBVS.

## Methods

### Bayesian variable selection with probit model

The binary probit model is incorporated to implement BVS method. Let us assume that (*y*, X) indicates the observed data, with *y*_*n *× 1 _a dichotomous categorical outcome vector coded as 1 or 0 representing for RA affected or RA unaffected, respectively, and X_*n *× *p *_the predictor matrix. Let *z *be an *n *× 1 vector of latent variables, while each *z*_*i*_, associated with a categorical outcome, *y*_*i*_, is described by a linear regression model:

*z*_*i *_= X_*i*_*β *+ *ε*_*i*_,   *ε *~ N(0, *σ*^2^),   *i *= 1,..., *n*.

The relationship between *z*_*i *_and *y*_*i *_is defined by *y*_*i *_= 1 if *z*_*i *_> 0 and *y*_*i *_= 0 otherwise. The likelihood function of the model defined in Eq. (1) may be written as *f*(*z*|*β*, *σ*):

*f*(*z*|*β*, *σ*) = N_n_(X*β*, *σ*^2^I).

The variable selection problem arises from the fact that it would be preferable to exclude some unknown subset of the predictors that have negligible influence on the outcome. Thus, statistical models for the variable selection problem can be represented by a selection vector, which is a set of binary indicator variables *γ *= (*γ*_1_,..., *γ*_*p*_), where *γ*_*j *_= 1 or 0 corresponds to inclusion or exclusion of predictor *j *in the model, respectively. The prior distribution of the model indicator variables, *π*(*γ*), is chosen to reflect prior belief in whether particular SNPs are associated with RA status in our case. A reasonable choice of the prior information might be to have the *γ*_*j *_(*j *= 1,..., *p*) independent with probability *π*(*γ*_*j *_= 1) = 1-*π*(*γ*_*j *_= 0) = *p*_*j*_, thus

*π*(*γ*) = ∏ *p*_*j*_^*γj *^(1-*p*_*j*_)^1-*γj*^.

The residual variance *σ*^2 ^for the *γ*^th ^model is modeled as a realization from an inverse gamma prior:

*π*(*σ*^2^|*γ*) = IG(*ν*/2, *νλ*_*γ*_)

which is equivalent to *νλ*_*γ *_~ *χ*_*ν*_^2^. Because the value of selection vector, *γ*, is of interest and is unknown, the uncertainty underlying variable selection can be modeled by a mixture prior:

*π*(*β*, *σ*, *γ*) = *π*(*β*|*σ*, *γ*) *π*(*σ*|*γ*) *π*(*γ*).

The posterior distribution of (*β*, *σ*, *γ*) can be obtained from the product of the likelihood function of the model in Eq. (2) and the prior defined in Eq. (5):

*π*(*β*, *σ*, *γ*|*z*) = *f*(*z*|*β*, *σ*) *π*(*β*|*σ*, *γ*) *π*(*σ*|*γ*) *π*(*γ*).

Therefore, integrating out *β *and *σ *from Eq. (6) yields the posterior distribution of the selection vector *γ*:

*π*(*γ*|*z*) ≈ g(*γ*) ≡ *π*(*γ*) ∫ *f*(*z*|*β*, *σ*) *π*(*β*|*σ*, *γ*) *π*(*σ*|*γ*) *π*(*γ*) *dβdσ*.

Based on this setting, Metropolis algorithm with Gibbs sampling was incorporated to sample (*γ*, *z*) as follows: 1) Metropolis step: *π*(*γ*|*z*) ≈ g(*γ*) with acceptance probability {g(*γ*^new^)/g(*γ*^old^), 1}; 2) (*z*|*γ*, X) has a truncated normal distribution.

In order to update each transition from *γ*^old ^to *γ*^new^, the Metropolis algorithm uses deletion, addition, and swapping moves discussed by Brown et al. [4]. Details of the prior information, the posterior distribution, and the updating procedure can be found in George and McCulloch [5] and Sha et al. [2].

### Iterative Bayesian variable selection

As mentioned, BVS uses long iterations and take a long time for the Metropolis algorithm to find suitable subset of SNPs. In the worst case, BVS might be unable to provide a promising subset. In order to overcome these problems, we propose to use BVS iteratively with a relatively small number of iterations, which is termed IBVS, to increase the speed of search for promising subsets of SNPs. There are two basic ideas behind IBVS. First, if *γ*_*j *_is not significant at the early stage of iteration when long iteration is incorporated in BVS, then the *j*^th ^marker is excluded in the final model. Second, the model that has high probability is more likely to appear at the early stage of iteration. From these facts, we can use BVS iteratively with relatively small number of iterations to increase the speed of searching for promising subsets of markers. This IBVS can be implemented by the following steps: 1) Start with BVS with full model, i.e., the model having all SNPs. 2) Choose a model for next iteration of BVS, e.g., the model that has highest posterior probability. 3) Repeat Step 1 and 2 with the model chosen in Step 2 until a certain number of SNPs remain in the model.

### Materials

There were 100 replicates in GAW15 Problem 3 data sets. Each replicate consisted of 1500 nuclear families (two parents and two offspring) that had an affected sibling pair (ASP) and 2000 unrelated control subjects that had no first-degree relatives with RA. Three marker sets were provided: 1) a set of 730 microsatellite markers fairly evenly spaced on chromosomes with an average intermarker distance of about 5 cM; 2) a set of 9187 SNPs distributed on the genome to mimic a 10 K SNP chip set; 3) a very dense map of 17,820 SNPs on chromosome 6. We utilized the second marker set for our analysis. According to the answer distributed by GAW15, there are three loci (DR, C, and D) on chromosome 6 that increase the risk of RA. Loci DR and C are located between SNP6_153 and SNP6_154, and are in complete linkage equilibrium. Locus C increases RA risk only in women. Locus D is located between SNP6_161 and SNP6_162, and has a rare minor allele frequency of 0.0083. We focused our analysis on the 674 SNPs on chromosome 6 to evaluate the IBVS method.

We first constructed three case-control panels for each of the 100 replicates. The first panel included both males and females. One affected offspring was randomly selected from each of the 1500 families that had an ASP. These 1500 unrelated affected subjects were used as cases. The 2000 unrelated control subjects were used as controls. In addition, because locus C increases RA risk only in women, we also constructed a female case-control panel and a male case-control panel. To maximize the number of female cases, we randomly selected one affected female offspring from each of the ASP families that had at least one female offspring. The female case-control panel consisted of ~1400 unrelated affected female offspring selected from the ASP families and ~1000 female controls. The male case-control panel was constructed similarly. It consisted of ~680 cases and ~1000 controls. Therefore, a total of three case-control panels (total, female, and male, respectively), each having 100 replicates, were constructed. This data set was named DS1.

Second, in order to evaluate the performance of IBVS when *p *» *n*, we constructed a subset case-control panel from each of the case-control panels in DS1 by randomly selecting 50 cases and 50 controls. The same 674 SNPs were kept in the panel. This data set was called DS2 (*n *= 100, *p *= 674).

Finally, in order to compare the performance between IBVS and traditional SVS directly, we selected a subset of 50 SNPs located between SNP6_128 and SNP6_177 from DS2. This dataset was named DS3 (*n *= 100, *p *= 50).

As a comparison, we also carried out the association analysis using BVS and SVS, which was implemented in the Proc Logistic procedure in SAS. We summarized all results obtained from IBVS, BVS, and SVS by calculating power for each SNP indicator, *γ*_*j*_, as follows:

$$\begin{array}{cc} {\text{Power}}_{j}=\frac{1}{n}\sum_{i=1}^{n}I(\gamma_{ij}=1), & j=1,...,p, \end{array}$$

where *I *is the indicator function satisfying *I*(*γ*_*ij *_= 1) = 1 if *γ*_*ij *_= 1 and *I*(*γ*_*ij *_= 1) = 0 otherwise; and *n *is the total number of replicates. Like other iterative methods, e.g., Newton method, there can be many stopping rules that can be applied in Step 3 in IBVS. We used the predetermined number of SNPs (10) based on empirical experience to stop the IBVS algorithm.

## Results and discussion

**Figure 1:**
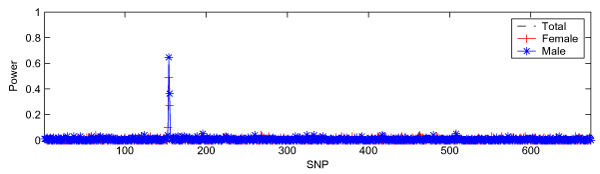
**IBVS in DS2**. All three panels in DS2 have 100 samples (50 cases and 50 controls) and 674 SNPs.

We examined the performance of IBVS when *p *» *n *by implementing the IBVS in DS2 and summarized the results in Figure 1. We found a peak corresponding to the genomic region where loci DR and C are located for all three panels (total, female, and male), demonstrating that IBVS properly identified two trait loci (DR and C). Figure 1 also shows that IBVS was unable to identify locus D. This is, however, not completely unexpected due to the fact that the minor allele frequency in locus D is very low (0.0083), and we have more predictors (674 SNPs) than samples (100).

**Figure 2:**
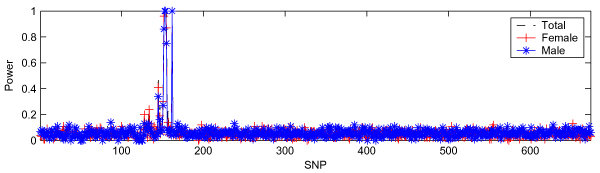
**SVS in DS1**. Total panel has 1500 cases and 2000 controls; female panel, ~1400 cases and ~1000 controls; and male panel, ~680 cases and ~1000 controls. All panels have 674 SNPs.

Figure 2 shows the results when applying SVS to DS1. For all three panels, SVS successfully identified three trait loci (DR, C, and D) with two high peaks. One peak corresponded to the region between SNP6_153 and SNP6_154, where loci DR and C are located, and the other corresponded to SNP6_162, where locus D is located. However, there were a few other SNPs with relatively high powers around SNP6_145, but those were apparently false positives.

**Figure 3:**
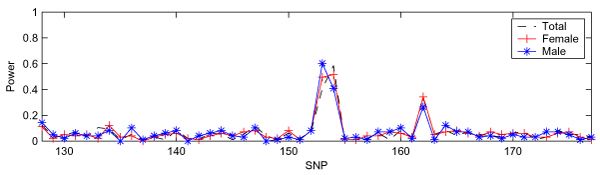
**IBVS in DS3**. All three panels in DS3 have 100 samples (50 cases and 50 controls) and 50 SNPs.

**Figure 4:**
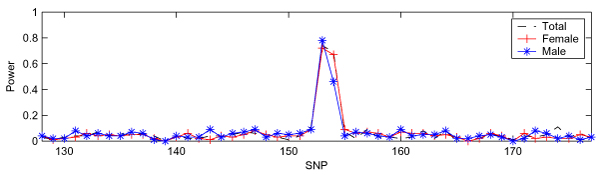
**SVS in DS3**. All three panels in DS3 have 100 samples (50 cases and 50 controls) and 50 SNPs.

Although we illustrated the validity of IBVS by comparing the results obtained from SVS, it was difficult to directly compare two search methods given that they were applied to two different data sets (100 samples of DS1 and ~3500 samples of DS2). In order to compare the performance between IBVS and SVS directly, we applied both methods to DS3, which focused on the SNPs between SNP6_128 and SNP6_177 (*n *= 100, *p *= 50). The results obtained from IBVS and SVS are shown in Figure 3 and Figure 4, respectively. Figure 3 shows that, for each of the three case-control panels, there are two separated peaks: one relatively high peak at SNP6_153 and SNP6_154 and the other at SNP6_162. This demonstrated that IBVS successfully identified three trait loci (DR, C, and D) including the one with a rare allele frequency. However, the results from SVS had only one peak corresponding to loci DR and C for all the three case-control panels (Figure 4). SVS was unable to identify locus D, which has a very small minor allele frequency. Therefore, we concluded that the performance of IBVS is better than that of SVS when *n *> *p*.

We also applied BVS to DS2 to compare the performance between IBVS and BVS. The results showed that the final model provided by BVS with 10,000 iterations and 5000 burn-in periods for each replicate contained over 300 SNPs, which demonstrated that BVS tends to yield more false positives. Therefore, IBVS improved the performance in variable selection as compared to BVS. In addition, the overall run time for BVS was about five times slower than that for IBVS.

With the goal of investigating sample size effect in IBVS, we applied IBVS to a data set, in which each case-control panel had five cases and five controls randomly selected from each case-control panel in DS1 and 50 SNPs between SNP6_128 and SNP6_177 (*n *= 10, *p *= 50). We found that IBVS identified the same two loci (DR and C), as when applied to DS2 (Figure 1), but was unable to identify Locus D, although the power was lower than that in Figure 1.

Another interesting question is how SNP density affects the performance of IBVS. We applied IBVS to another data set in which each case-control panel again consists of ten cases and controls (five each) randomly selected from each of case-control panels in DS1, but the 50 SNPs were selected in a wide genomic region (between SNP6_104 and SNP6_203) by selecting every other SNP. With this data set, we were able to identify SNP6_154 with a slightly higher power as compared to that with a denser SNP map. The likely reason for this is that the between-variable correlation included in the model has an effect on the performance of the method. When the SNPs are relatively loosely distributed, the LD (between-variable correlation) among them is lower and IBVS performs better. However, this does not mean we will be able to identify a disease mutation with very loosely distributed SNPs. The success of a genome-wide association study still relies on whether a marker in high LD with the disease mutation is included in the study set of SNPs.

## Conclusion

We applied the IBVS method to the case-control data constructed from the simulated RA data sets of GAW15. When the number of sample size (100 observations) is larger than the number of predictors (50 SNPs), i.e., *n *> *p*, we were able to identify association with RA status on chromosome 6 at the location where loci DR and C are located by both IBVS and SVS. However, the association between RA status and locus D was identified only by IBVS. With a small sample size of 100 and large number of predictors (674 SNPs), i.e., *n *» *p*, IBVS can still identify association with RA status on chromosome 6 at the location of Loci DR and C. We concluded that IBVS method is promising for identifying genetic determinants in genome-wide association studies when the number of genetic markers is much larger than the number of samples.

## Competing interests

The author(s) declare that they have no competing interests.

## References

[B1] West M, Nevins JR, Marks JR, Spang R, Zuzan H (2000). DNA microarray data analysis and regression modeling for genetic expression profiling. Discussion Paper 00-15.

[B2] Sha N, Vannucci M, Tadesse M, Brown P, Dragoni I, Davies N, Roberts T, Contestabile A, Salmon M, Buckley C, Falciani F (2004). Bayesian variable selection in multinomial probit models to identify molecular signatures of disease stage. Biometrics.

[B3] George E, McCulloch R (1993). Variable selection via Gibbs sampling. J Am Stat Assoc.

[B4] Brown P, Vannucci M, Fearn T (1998). Multivariate Bayesian variable selection and prediction. J Roy Stat Soc Series B.

[B5] George E, McCulloch R (1997). Approaches for Bayesian variable selection. Stat Sinica.

